# Inhibitory Effect of Chinese Propolis on Phosphatidylcholine-Specific Phospholipase C Activity in Vascular Endothelial Cells

**DOI:** 10.1155/2011/985278

**Published:** 2010-10-24

**Authors:** Hongzhuan Xuan, Ruiliang Zhu, Yajing Li, Fuliang Hu

**Affiliations:** ^1^College of Animal Sciences, Zhejiang University, Hangzhou 310029, China; ^2^School of Life Science, Liaocheng University, Liaocheng 252059, China; ^3^College of Animal Science and College of Veterinary Medicine, Shandong Agricultural University, Taian 271018, China

## Abstract

To understand the mechanisms underlying the anti-inflammatory action of Chinese propolis, we investigated its effect on the activity of phosphatidylcholine-specific phospholipase C (PC-PLC) that plays critical roles in control of vascular endothelial cell (VEC) function and inflammatory responses. Furthermore, p53 and reactive oxygen species (ROS) levels and mitochondrial membrane potential (Δ*ψ*m) were investigated. Our data indicated that treatment of Chinese propolis 6.25 and 12.5 *μ*g/ml for 12 hours increased VEC viability obviously. Exposure to Chinese propolis 6.25, 12.5, and 25 *μ*g/ml for 6 and 12 hours significantly decreased PC-PLC activity and p53 level, and ROS levels were depressed by Chinese propolis 12.5 *μ*g/ml and 25 *μ*g/ml dramatically. The Δ*ψ*m of VECs was not affected by Chinese propolis at low concentration but disrupted by the propolis at 25 *μ*g/ml significantly, which indicated that Chinese propolis depressed PC-PLC activity and the levels of p53 and ROS in VECs but disrupted Δ*ψ*m at a high concentration.

## 1. Introduction

Propolis is a resinous substance collected by honeybees from the bud and bark of certain trees and plants. It has been used in folk medicine from ancient times in many countries. Recently, it has been reported to possess various biological activities, such as antibacterial [1], antiviral [2, 3], anti-inflammatory [4, 5], anticancer [6, 7], antioxidant [8, 9], and antiangiogenesis activities [10]. Therefore, propolis has been extensively used in food and beverages to improve health and prevent diseases such as inflammation, diabetes, heart disease and cancer [11–13].

In recent years, the anti-inflammatory action of propolis has been widely documented [14–16], and we also reported that Chinese propolis exhibited significant anti-inflammatory effects in animal models with respect to thoracic capillary vessel leakage in mice, carrageenan-induced oedema, carrageenan-induced pleurisy, and acute lung damage in rats [5]. However, the molecular mechanisms underlying such protect effects of Chinese propolis have not been fully elucidated. 

Phosphatidylcholine-specific phospholipase C (PC-PLC), an important member of phospholipase C family, hydrolyzes phosphatidylcholine to produce phosphocholine and diacylglycerol in a number of receptor-stimulated cells. Phosphocholine and diacylglycerol are the most important second messengers that have been implicated in a wide range of cellular response such as cell growth, differentiation, senescence, and apoptosis of mammalian cells [17, 18]. Previous research has demonstrated that PC-PLC plays critical roles in various inflammatory responses [19–21]. Furthermore, a recent study found that PC-PLC contributes to the progression of atherosclerosis, which is considered to be a chronic inflammatory disease. Pharmacological blockade of PC-PLC activity by D609 inhibited the progression of preexisting atherosclerotic lesions in apoE^−/−^ mice; as well, inhibition of PC-PLC activity in vascular endothelial cells (VECs) reduced the expression of vascular cell adhesion molecule-1, intercellular adhesion molecule-1, and monocyte chemotactic protein-1 induced by oxidized low-density lipoprotein contributing to the progression of atherosclerosis [22].

However, the effect of Chinese propolis on PC-PLC activity in VECs deprived of basic fibroblast growth factor (FGF-2) and serum, which leads to inflammation in the vessel wall, is not known. Considering the important roles of PC-PLC in endothelial apoptosis and its proinflammatory properties, we investigated whether Chinese propolis (6.25, 12.5, and 25 *μ*g/mL) affected PC-PLC activity in VECs deprived of FGF-2 and serum. Furthermore, we investigated the effects of Chinese propolis on p53 and reactive oxygen species (ROS) levels and mitochondrial membrane potential which were regulated by PC-PLC in VECs.

## 2. Materials and Methods

### 2.1. Materials

Chinese propolis was obtained from colonies of honeybees, *A. mellifera* L., in Shandong province of north China, and the main plant origin was poplar (*Populus* sp.). Medium 199 and fetal bovine serum (FBS) were from Hyclone Lab Inc. (USA). FGF-2 was from EssexBio Group, China. L-*α*-phosphatidylcholine, sulforhodamine B (SRB), and 2′,7′-dichlorodihydrofluorescin (DCHF) were from Sigma Co. (USA). JC-1 was from Invitrogen (USA). Primary antibodies against p53, *β*-actin, and secondary antibody (horseradish peroxidase) were from Santa Cruz Biotechnology (USA). All other reagents were ultrapure grade.

### 2.2. Preparation of Propolis Extracts

 Propolis samples were extracted with ethanol at room temperature for 24 hours The ethanol suspension was filtered under reduced pressure, and the filter liquid was then concentrated in a rotary evaporator under reduced pressure at 40°C until reaching a constant weight and then redissolved in ethanol. The prepared propolis was stored under a dry condition at 4°C.

### 2.3. Cell Culture

 Human umbilical vein endothelial cells (HUVECs) were obtained by using the method of Nachman and Jaffe [23]. HUVECs were cultured in M199 medium supplemented with 20% FBS and 70 ng/mL FGF-2. Incubation was carried out in a humidified atmosphere with 5% CO_2_ at 37°C. The cells were seeded on plates coated with 0.1% gelatin and allowed to grow to confluence before experiments.

### 2.4. Exposure of VECs to Chinese Propolis

When the VEC cultures reached confluence, the medium was replaced with basal M199 medium (without FBS and FGF-2). Then the cells were divided for treatment: (a) culture in M199 medium with FBS and FGF-2 (normal), (b) deprived of serum and FGF-2 but cultured in M199 medium with ethanol at <0.1% (v/v) (control), and (c) culture in basal M199 medium with Chinese propolis (6.25, 12.5, and 25 *μ*g/mL). Chinese propolis was dissolved in ethanol, with final concentration of ethanol in the culture medium <0.1% (v/v). Ethanol at 0.1% (v/v) did not affect cell viability. The morphological changes of cells were observed under a phase contrast microscope (Nikon, Japan) at 6, 12, and 24 hours, respectively.

### 2.5. Cell Viability Assay

VECs were seeded in 96-well cell culture plates and grown to confluence then washed once with basal M199 medium. Then the cells were deprived of serum and FGF-2 and treated with Chinese propolis (6.25, 12.5 and 25 *μ*g/mL). At 6, 12, and 24 hours, cells were precipitated for 1 hour at 4°C with 100 *μ*L 10% trichloroacetic acid and stained with SRB. The optical density was measured at 540 nm after reconstitution of the dye in 100 *μ*L 10 mM Tris base [24]. The viability (%) was expressed as (OD of treated group/OD of control group) ×100%. The viability of the control group was set at 100%.

### 2.6. PC-PLC Activity Assay

PC-PLC activity assay was performed as the described methods in [25, 26]. In brief, we prepared the enzyme and used L-*α*-phosphatidylcholine as the substrate of PC-PLC. The optical density was measured at 660 nm. Enzyme activity was expressed as nanomoles per minute per milligram protein.

### 2.7. Western Blot Analysis

Western blot assay of p53 level was performed as previously described in [27]. Twenty micrograms of protein was separated by 15% SDS-PAGE and transferred onto PVDF membrane. The relative quantities of the proteins were evaluated by use of Quantity one software.

### 2.8. Measurement of Reactive Oxygen Species (ROS) Production

The effect of Chinese propolis on ROS production in VECs was determined by use of a fluorescent probe, DCHF, which can be oxidized into fluorescent dichlorofluorescein (DCF) by intracellular ROS [28]. After treating cells with different concentrations of Chinese propolis for 6, 12, and 24 hours, cells were incubated with DCHF for 30 minutes at 37°C. Then cells were washed with basal M199 medium 3 times then observed on laser scanning confocal microscopy. The level of ROS was quantified by the software for the laser scanning confocal microscope. Results were shown as relative fluorescence intensity ratio to that of the control.

### 2.9. Measurement of Mitochondrial Membrane Potential

To measure mitochondrial membrane potential, the fluorescent dye JC-1 was used. JC-1 exists as a monomer at low mitochondrial membrane potential and emits green fluorescence but forms aggregates and emits red fluorescence at high mitochondrial membrane potential [29]. After treating cells with different concentrations of Chinese propolis for 6, 12, and 24 hours, cells were incubated with JC-1 for 15 minutes at 37°C. Then cells were washed with basal M199 medium 3 times then observed on laser scanning confocal microscopy. The mitochondrial membrane potential was quantified by use of the software of the laser scanning confocal microscope. Results were shown as ratio of red to green fluorescence.

### 2.10. Statistical Analysis

All experiments were performed in duplicate and repeated at least 3 times. Data are expressed as means ± SD. Statistical analysis involved use of the paired *t* test by SPSS v11.5 (SPSS Inc., Chicago, IL). A *P* < .05 was considered statistically significant.

## 3. Results

### 3.1. Effect of Chinese Propolis on VEC Viability

**Figure 1:**
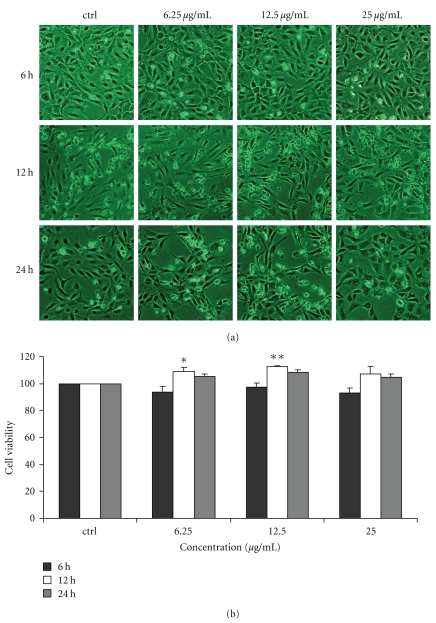
Effect of Chinese propolis on cell viability. VECs were treated with Chinese propolis 6.25, 12.5, and 25 *μ*g/mL for 6, 12, and 24 hours, respectively. ctrl: control group. (*P < .05, **P < .01 versus control group, *n* = 3).

After treated with Chinese propolis 6.25, 12.5, and 25 *μ*g/mL for 6, 12, and 24 hours, the cell viability was examined by SRB assay. At 12 hours, Chinese propolis 6.25, and 12.5 *μ*g/mL significantly increased VEC viability (*P < .05, **P < .01; Figure 1), and the propolis 25 *μ*g/mL had no significant effect on cell viability.

### 3.2. Effect of Chinese Propolis on PC-PLC Activity

**Figure 2:**
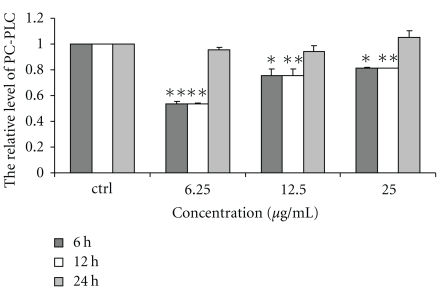
Effect of Chinese propolis on the activity of PC-PLC. VECs were treated with Chinese propolis 6.25, 12.5, and 25 *μ*g/mL for 6, 12, and 24 hours, respectively. ctrl: control group. (*P < .05, **P < .01 versus control group, *n* = 3).

 The activity of PC-PLC in VECs was significantly depressed by Chinese propolis 6.25, 12.5, and 25 *μ*g/mL at 6 and 12 hours (*P < .05, **P < .01; Figure 2). But, the propolis had no effect on PC-PLC activity at 24 hours.

### 3.3. Effect of Chinese Propolis on p53 Level

**Figure 3:**
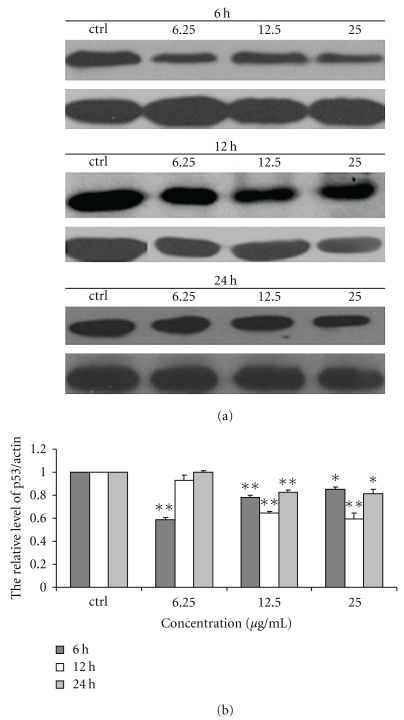
Effect of Chinese propolis on the level of p53. (a) The level of p53 was detected by western blot analysis at 6, 12 and 24 hours. ctrl: control group. (b) The hemiquantification of p53 level. (*P < .05, **P < .01 versus control group, *n* = 3).

Chinese propolis 6.25 *μ*g/mL decreased the level of p53 protein at 6 hours significantly (**P < .01), and the propolis 12.5 and 25 *μ*g/mL also depressed p53 level at 6, 12, and 24 hours (*P < .05, **P < .01; Figure 3).

### 3.4. Effect of Chinese Propolis on ROS Level

**Figure 4:**
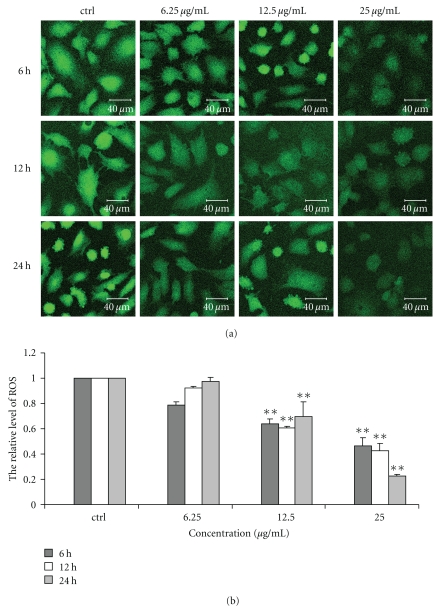
Effect of Chinese propolis on ROS level. (a) Fluorescent micrographs obtained at 6, 12, and 24 hours. ctrl: control group. (b) The relative quantity of ROS level in VECs (**P < .01 versus control group, *n* = 3).

Chinese propolis 12.5 *μ*g/mL significantly decreased ROS level in VECs at 6, 12 and 24 hours as compared with the control group (**P < .01). It significantly decreased the level of ROS at 25 *μ*g/mL from 6 to 24 hours (**P < .01). However, Chinese propolis 6.25 *μ*g/mL had no effect on ROS level (Figure 4).

### 3.5. Effect of Chinese Propolis on Mitochondrial Membrane Potential

**Figure 5:**
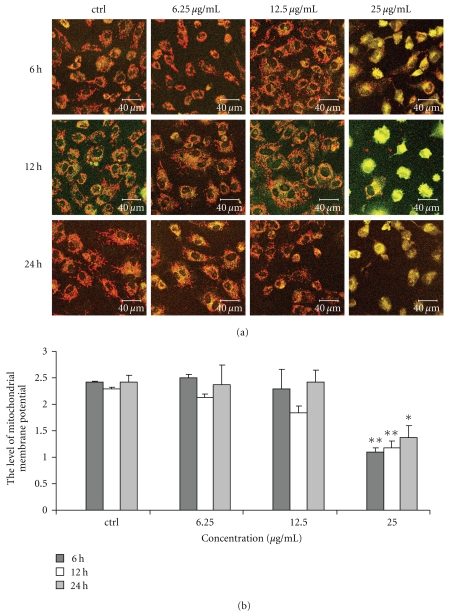
Changes in mitochondrial membrane potential caused by Chinese propolis. (a) Fluorescent micrographs obtained at 6, 12, and 24 hours. ctrl: control group. (b) The relative quantity of mitochondrial membrane potential in VECs (*P < .05, **P < .01 versus control group, *n* = 3).

We used the lipophilic cation JC-1 to evaluate the effect of Chinese propolis on mitochondrial membrane potential in VECs. Chinese propolis at 6.25 and 12.5 *μ*g/mL had no significant effect on the membrane potential (Figure 5); however, the propolis 25 *μ*g/mL decreased the mitochondrial membrane potential significantly (*P < .05, **P < .01).

## 4. Discussion

Propolis usually contains a variety of chemical compounds, such as polyphenols (flavonoids, phenolic acids, and their esters), terpenoids, steroids, and amino acid. The composition of propolis depends on the vegetation at the site of collection. Its chemical constituents and antioxidant activities of Chinese propolis have been studied in detail [30]. In addition Chinese propolis has been extensively used in food and beverages to improve health and prevent diseases such as inflammation, diabetes, heart disease, and cancer [11–13]. However, the molecular mechanisms underlying such effects of Chinese propolis have not been fully elucidated. In this study, the results showed that Chinese propolis depressed PC-PLC activity and the levels of p53 and ROS in VECs; however, disrupted mitochondrial membrane potential at a high concentration. These findings provide new evidence for understanding the mechanism underlying Chinese propolis's anti-inflammatory action. Moreover, the data suggest that Chinese propolis at a higher concentration may disrupt mitochondrial membrane potential of VECs, which is not well for vascular system.

PC-PLC is a key upstream signal molecule. Miao et al. previous studies showed that PC-PLC played a significant role during VEC senescence and apoptosis [18, 31]. VEC impairment of senescence and apoptosis leads to enhanced vessel wall permeability to cytokines, growth factors, lipids and immune cells, increases coagulatory activity of VEC, and induces atherosclerotic plaque rupture [32]. Atherosclerosis is considered to be a chronic inflammatory disease. In the present study, we found that the activity of PC-PLC significantly decreased in cells treated with Chinese propolis. The decrease in PC-PLC activity might be involved in the protect effect of Chinese propolis in VECs deprived of FGF-2 and serum.

p53 is another key protein in VEC associated with the activity of PC-PLC and plays an important role in VEC apoptosis signal transduction pathways. Apoptosis induced by p53 is firmly established as a central mechanism of tumour suppression [33]. Cheng et al. reported that PC-PLC possibly mediated the induction of apoptosis by cooperation with ROS and p53 [17]. Here, we found that p53 level was markedly suppressed in the cells treated with Chinese propolis, which was accompanied by decreased activity of PC-PLC. 

ROS are normally generated in the mitochondria and have been identified as important mediators that regulate signal transduction [34, 35]. As well, mitochondria plays important role in energy metabolism and regulation of cell death. These roles, in turn, seem to be intimately linked to the role of the factors as the major intracellular source of ROS [36]. Previous reports showed that apoptosis occurred when intracellular ROS was elevated. Here, we found that the level of ROS in the cells treated with Chinese propolis significantly decreased. Moreover, the level of ROS in the cells treated with Chinese propolis 25 *μ*g/mL was excessively decreased from 6 to 24 hours; at the same time, mitochondrial membrane potential was decreased, which indicated that excessive decrease of ROS disrupted mitochondrial membrane potential. Thus, the balance of intracellular ROS might be important in cell survival. Very low level of ROS might have a harmful effect as very high level of ROS. And Chinese propolis preparation used may not contain any significant amounts of glucose oxidase that generates H_2_O_2_, which can role on to generate hydroxyl radical, and several of the polyphenols present in our preparation may, like quercetin, be taken up by cells which could affect the fluorescence data.

**Figure 6:**
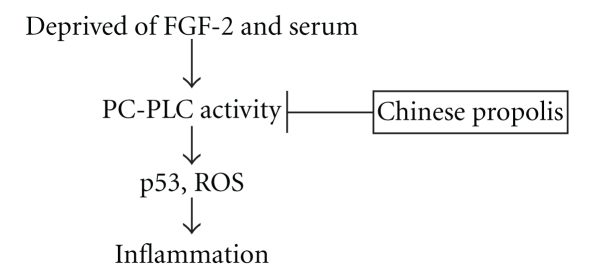
Schematic diagram of inhibitory effect of Chinese propolis on PC-PLC activity in vascular endothelial cells deprived of FGF-2 and serum. Chinese propolis depressed PC-PLC activity and further regulated p53 and ROS levels to play anti-inflammatory action.

To our knowledge, this is the first report to reveal the effect of Chinese propolis on PC-PLC activity in VECs. Our present results showed that one major mechanism underlying the effect of Chinese propolis on PC-PLC activity might be through regulating p53 and ROS (Figure 6). PC-PLC might serve as a marker of anti-inflammat effects of propolis in the future. Further, in modulating PC-PLC activity and the levels of p53 and ROS, the effect of Chinese propolis 12.5 *μ*g/mL was more effective than that of 6.25 *μ*g/mL. Chinese propolis at high concentrations might be harmful, so it should be used at safe and effective doses according to different situations. We would like to further investigate the anti-inflammatory effects of mixtures of polyphenols known to be present on propolis.
